# Unraveling Drug Penetration of Echinocandin Antifungals at the Site of Infection in an Intra-abdominal Abscess Model

**DOI:** 10.1128/AAC.01009-17

**Published:** 2017-09-22

**Authors:** Yanan Zhao, Brendan Prideaux, Yoji Nagasaki, Min Hee Lee, Pei-Yu Chen, Landry Blanc, Hsinpin Ho, Cornelius J. Clancy, Minh Hong Nguyen, Véronique Dartois, David S. Perlin

**Affiliations:** a Public Health Research Institute, New Jersey Medical School, Rutgers Biomedical and Health Sciences, Newark, New Jersey, USA; b Department of Medicine, Division of Infectious Diseases, University of Pittsburgh School of Medicine, Pittsburgh, Pennsylvania, USA

**Keywords:** intra-abdominal candidiasis, echinocandin, drug penetration, matrix-assisted desorption ionization (MALDI) mass spectrometry imaging

## Abstract

Intra-abdominal candidiasis (IAC) is a prominent invasive fungal infection associated with high mortality. Prompt antifungal therapy and source control are crucial for successful treatment. Echinocandin antifungal drugs are first-line agents; however, their clinical effectiveness is highly variable, with known potential for breakthrough resistance, and little is known about drug exposure at the site of infection. Using matrix-assisted desorption ionization mass spectrometry imaging technology, we investigated the spatial and quantitative distribution in tissue lesions for two echinocandin drugs, micafungin and CD101, in a clinically relevant IAC mouse model. Drug accumulation within lesions was observed with both drugs at their humanized therapeutic doses. CD101, but not micafungin, accumulated in lesions at levels above the mutant prevention concentration of the infecting strain. These findings indicate that current echinocandin drugs are limited by penetration at the site of infection and have implications for clinical outcomes and emergence of resistance in patients with IAC.

## INTRODUCTION

Candidemia and intra-abdominal candidiasis (IAC) are the two most common types of invasive candidiasis that have an associated high mortality (1–3). However, unlike candidemia, which has been the focus of most studies of invasive candidiasis, IAC is poorly understood (4). Scattered abscesses and microlesions are the predominant histopathological findings within abdominal organs from humans with IAC. Prompt source control and institution of antifungal therapy are major determinants of successful outcomes among patients with IAC (2, 4). It has been postulated that restricted drug penetration into abscesses is the main cause of antifungal treatment failure and creates a hidden reservoir of resistance (5).

Echinocandins, the first class of antifungals to target the fungal cell wall (6), are recommended as first-line therapies for most types of invasive candidiasis (7, 8), but treatment failures occur in up to 40% of cases (9). In addition, despite the overall relatively low resistance frequency, widespread and expanding echinocandin usage has led to the emergence of resistance, particularly among Candida glabrata strains (10–12). Limited data have suggested that echinocandin delivery to infection sites is often insufficient to achieve concentrations that eliminate Candida or suppress resistance (13, 14), which may account for a considerable amount of treatment failures. However, data on the infection site pharmacokinetics (PK) of echinocandins are extremely scarce and nothing is known about penetration into tissue lesions, although one population PK study reported that micafungin exposure in peritoneal fluid was significantly lower than that in plasma in IAC patients (14).

Sufficient penetration into infected tissue compartments is a key requirement for efficacy of all antimicrobial agents (15, 16). Taking the specific histopathology of IAC into account, perhaps the most clinically important and informative data are how a drug distributes and penetrates into abscesses or other infected lesions within tissues rather than drug concentrations in serum or whole organs. Matrix-assisted laser desorption ionization mass spectrometry imaging (MALDI-MSI) has emerged as a powerful tool to acquire spatially allocated molecular information about drug distributions in tissues (17, 18). MALDI-MSI has been used increasingly in drug discovery and development because of its ability to provide spatial distribution of target compounds without the need for special labels (19), as well as potential biomarkers of efficacy and toxicity (20, 21). Here, we took the initiative to apply this technology as well as standard analytical techniques to investigate echinocandin drug penetration at the site of infection in a clinically relevant IAC mouse model involving C. albicans (22).

Micafungin is a widely prescribed echinocandin drug used as a standard of care for patients with suspected or confirmed IAC. CD101 (Cidara Therapeutics, Inc.) is a next-generation echinocandin agent in clinical development that features exceptional stability and long half-life PK properties relative to those of other drugs in this class (23, 24). These properties allow a single dose to be administered safely at a much higher level than conventional echinocandin drugs (25). The objective of this study was to critically evaluate the penetration of echinocandin drugs at the site of infection and assess whether drug levels in lesions help account for the observed clinical response and potential for late-stage resistance emergence.

## RESULTS

### Tissue distribution and penetration after a single dose of micafungin and CD101. (i) Histopathology and MALDI imaging analysis.

**FIG 1:**
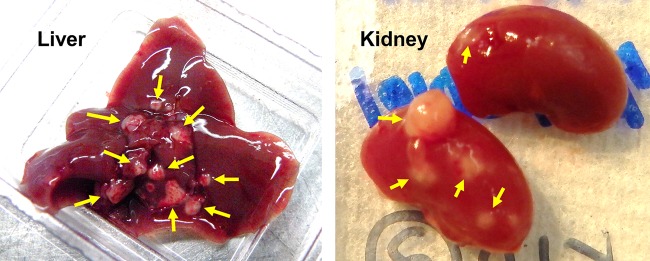
Liver and kidney abscesses at necropsy on day 3 postinfection. Neon yellow arrows point out multiple lesions formed in liver (left) and kidney (right).

The intra-abdominal candidiasis model yielded abundant heterogeneous lesions (Fig. 1) at 3 days postinfection. Echinocandin antifungal drugs then were introduced and the spatial distribution of micafungin and CD101 was visualized by MALDI-MSI, delivering high-resolution heat maps of drug concentrations in liver and kidney tissues. Side-by-side comparison of these images with histopathological staining (hematoxylin and eosin [H&E] and Gomori methenamine silver [GMS]) of adjacent sections revealed drug penetration in different lesion structures, as well as the relationship between drug distribution and location of fungal cells. Upon histopathological analysis, we found that lesions formed in abdominal organs are characterized by large macrophage/neutrophil infiltrates surrounding a necrotic core of various sizes. Fungus-specific GMS staining further supported that fungal load in lesions appeared to correlate with the necrotic severity of lesions. High-level fungal staining was observed predominantly in necrotic areas (see Fig. S1 in the supplemental material).

**FIG 2:**
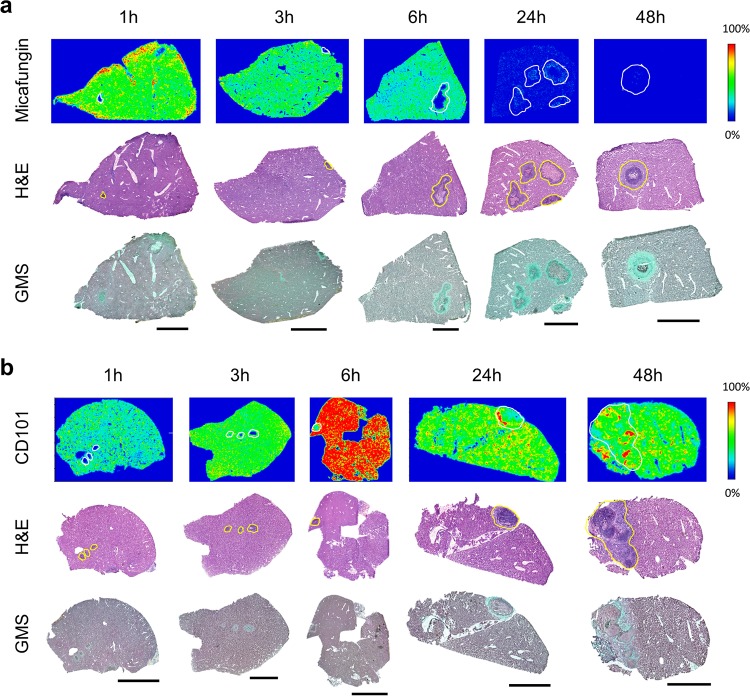
Drug distribution in infected liver tissues after single doses of micafungin and CD101. (a, upper) Ion maps of micafungin in representative liver tissues collected at 1, 3, 6, 24, and 48 h after a single dose of micafungin at 5 mg/kg. The signal intensity color bar is fixed for micafungin, with gradually increased intensity from blue (no signal) to red (max signal). H&E and GMS staining of adjacent sections are shown below each set of ion maps. Outlines highlight the lesion area on each tissue section. Scale bars, 3 mm. (b, upper) Ion maps of CD101 in representative liver tissues collected at 1, 3, 6, 24, and 48 h after a single dose of CD101 at 20 mg/kg. The signal intensity color bar is fixed for CD101, with gradually increased intensity from blue (no signal) to red (maximum signal). Matched H&E and GMS staining results are shown in the middle and bottom rows, respectively. Scale bars, 3 mm.

**FIG 3:**
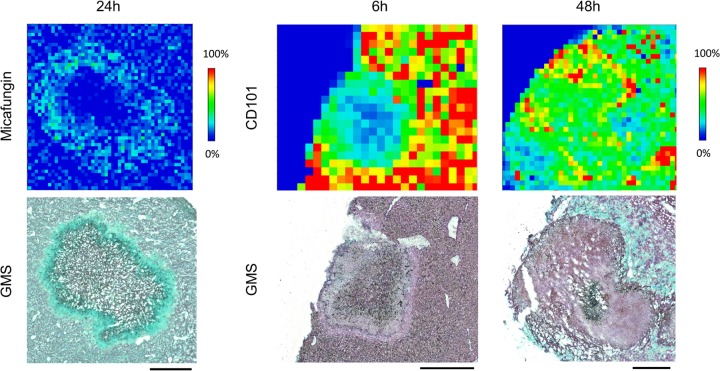
Close-up examination of drug penetration for micafungin at 24 h and CD101 at 6 and 48 h after single dosing. An enlarged view of drug distribution in a single lesion at pixel level is shown. Matched GMS staining of adjacent sections was placed on the bottom. Signal intensity is fixed for CD101 and micafungin. Scale bars, 5 mm.

After a single humanized dose, both echinocandins were quickly distributed into liver and kidney. However, the pharmacokinetics of tissue exposure and the pattern of lesion penetration were notably different for these two drugs. After a single-dose administration of micafungin at 5 mg/kg of body weight, drug quickly distributed into liver tissues and reached peak intensity at 1 h (Fig. 2a). Decreased drug intensities (relative drug abundance) over the entire tissue were observed at 3 h and 6 h, although drug signal was barely detectable in lesions until 6 h, when the drug was observed at the edge of the lesion with little detected penetration into the necrotic core. Penetration of the drug into the necrotic lesion was clearly observed at 24 h, when the micafungin signal was detected inside the lesion with noticeably higher intensities in the outer rim of the lesion than the necrotic center, as well as in the surrounding uninvolved tissue. An enlarged view of the 24-h MALDI image and the adjacent GMS-stained section (Fig. 3) showed that micafungin resided predominantly in the lesion edge, whereas fungal cells constitute a massive network throughout the lesion. Thus, interaction between the drug and fungi was limited in the outer part of the lesion, but within the necrotic center, where the majority of the fungal population resides, there was no detectable drug exposure. (The limit of detection [LOD] for MALDI-MSI analysis of micafungin and CD101 was 500 ng/g and 1 μg/g of liver or kidney tissue. Spot testing was performed for each drug to determine the LOD as previously described [19].) Moreover, without further dosing past 24 h, such drug retention within the lesion was too low to outcompete the quick drug clearance from liver, where drug levels dropped far below the limit of detection and no drug signals were detected at 48 h. In kidneys, the same kinetic pattern of tissue distribution and lesion penetration was observed for micafungin (Fig. S2a).

In liver, CD101 signal intensity was readily detected at the earliest time point investigated (1 h postdose) and steadily increased at 3 h and 6 h, with the highest drug signal detected at 6 h postdose (Fig. 2b). Thereafter, CD101 drug intensity slowly declined but still persisted strongly, even at 48 h postdose. Closer examination of the ion map and GMS staining revealed detectable lesion penetration (CD101 signal appeared inside lesions) as early as 3 h postdose, and a gradient of drug distribution was observed within the lesion at 6 h, with higher drug intensity in the outer area and fewer signals in the necrotic center (Fig. 3). At later time points (24 h and 48 h), CD101 was persisting with slow accumulation primarily within the lesion, while surrounding tissue drug levels were declining. The 48-h enlarged view mapped out a more balanced distribution of CD101 with drug signals detected throughout the necrotic lesion (Fig. 3). Drug distribution and penetration of CD101 in kidneys were similar to what was observed in liver, whereas lesion formation and histopathology in kidneys (Fig. S2b) was not as consistent as what was observed in livers with heterogeneous manifestations ranging from very tiny lesions to big or multifocal lesions.

### (ii) Quantitative evaluation of drug exposure in liver lesions.

**FIG 4:**
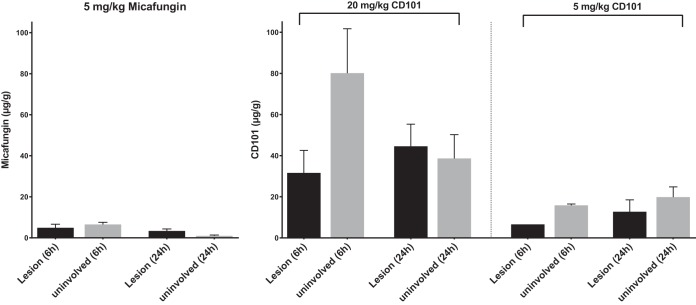
Quantification of drug exposure in liver lesions and surrounding tissues. Drug concentration was measured in lesions and surrounding uninvolved tissues dissected from liver sections collected at 6 h and 24 h after a single dose of micafungin at 5 mg/kg or CD101 at 20 and 5 mg/kg. Error bars show means ± standard deviations (SD) from 3 to 5 liver pieces or distinct lesions.

MALDI imaging analysis provides valuable information on spatial distribution and lesion penetration. However, it is only semiquantitative and not a measure of exact drug exposure at the site of infection. Hence, we next applied laser capture microdissection (LCM) followed by high-pressure liquid chromatography coupled to tandem mass spectrometry (LC-MS/MS) to quantify absolute drug concentrations in distinct compartments of involved tissues at two representative time points, 6 h and 24 h postdose. Only liver samples were analyzed due to the fact that kidney lesions were too small to dissect sufficient material to meet the LOD for LC-MS/MS analysis. Upon quantification (Fig. 4), the single dose of 5 mg/kg micafungin (at which dosage experimental serum drug levels ranged from approximately 7 to 10 μg/g at 6 h and around 2 μg/g at 24 h [26, 27]) resulted in drug retention at 6.5 and 1 μg/g in uninvolved surrounding tissues and 4.9 and 3.4 μg/g in lesions at 6 h and 24 h, respectively. In contrast, remarkably high levels of CD101 were found in liver tissues. After therapeutic dosing (20 mg/kg) of CD101 (which resulted in serum drug levels of 43 and 22 μg/g at 6 h and 24 h, respectively [24]), the average drug level at 6 h was 80.1 μg/g in the nonlesion portion and 31.6 μg/g in lesions. At 24 h, the drug level in surrounding tissue dropped significantly compared to the level at 6 h (*P* = 0.01) but were still high, with a mean concentration of 38.7 μg/g. Moreover, the mean drug concentration within lesions increased to 44.5 μg/g at 24 h, even though the statistical significance of such an increase was not achieved due to the small sample size. When the lower dose of CD101 (5 mg/kg) was administered, a proportionally decreased but high level of drug was observed from both compartments at both time points. Drug concentration was 15.8 μg/g at 6 h and 19.8 μg/g at 24 h in surrounding tissues and 6.6 and 12.7 μg/g in lesions at 6 h and 24 h, respectively.

### (iii) Liver burden assessment.

**FIG 5:**
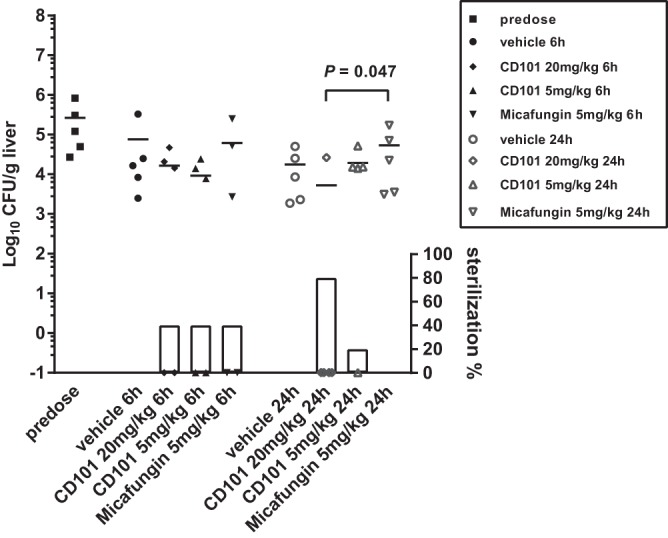
Liver burden comparison at 0 (predose), 6, and 24 h after single-dose treatment with CD101 at 20 or 5 mg/kg, micafungin at 5 mg/kg, and vehicle control. Each symbol represents liver burden of a single animal. Short lines are mean burdens determined for 5 mice in each treatment group. Symbols on the *x* axis represent mice with no liver burden (sterilization). The percentages of mice with liver sterilization were plotted as bars on the right *y* axis underneath burdens of each corresponding group. Compared to micafungin, the dose of 20 mg/kg CD101 resulted in significant burden reduction in liver at 24 h postdose, with a *P* value of 0.047, as well as in the percentage of sterilized animals.

To understand the relationship between tissue drug exposure and efficacy, we measured liver burdens at 0, 6, and 24 h after single-dose treatment of CD101 at 20 or 5 mg/kg and micafungin at 5 mg/kg (Fig. 5). An average burden of 5.4 log_10_ CFU/g was recovered from livers at day 3 postinfection before treatment. Mean burden counts at 6 h postdose were 4.9, 4.2, 4.0, and 4.8 log_10_ CFU/g for vehicle control, 20 mg/kg CD101, 5 mg/kg CD101, and 5 mg/kg micafungin, respectively. Although the burden reduction resulting from echinocandin treatment was not statistically significant, it is noteworthy that at 6 h no liver burden (effective sterilization) was observed in 2 out of 5 mice in each echinocandin treatment group but not in the vehicle control. By 24 h postdose, liver burdens showed an average of 4.3 log_10_ CFU/g for vehicle control, 3.7 and 4.3 log_10_ CFU/g for 20 and 5 mg/kg CD101, and 4.7 log_10_ CFU/g for 5 mg/kg micafungin-treated mice. Mice treated with 20 mg/kg CD101 had significantly lower liver burdens than mice treated with micafungin (*P* = 0.047), largely due to the fact that liver sterilization was achieved in 4 out of 5 mice in the 20-mg/kg CD101 group but none from the micafungin group. The CD101 5-mg/kg treatment also led to liver sterilization in one mouse, yet burden counts in the rest of the mice from the same group were not much different from that of either the vehicle control or micafungin treatment group.

### Comparison of drug accumulation at site of infection after multiple doses of micafungin and single dose of CD101. (i) MALDI imaging.

**FIG 6:**
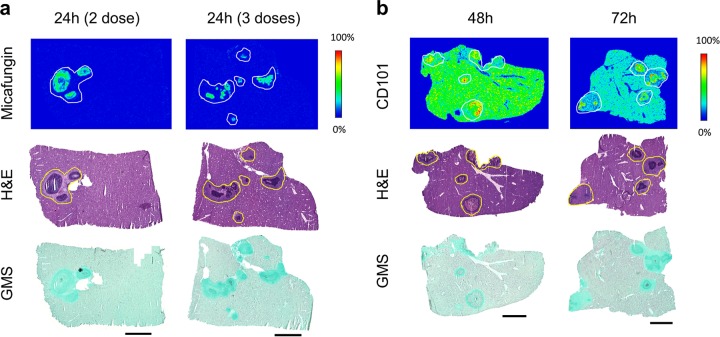
Drug penetration after multiple doses of micafungin (a) and single doses of CD101 (b). Micafungin is steadily accumulated in abscesses upon 2 and 3 doses. Micafungin signal was only detected from lesion centers at steady state (after 3 doses). CD101 diffused into lesions thoroughly at 48 h after single dosing and accumulated in necrotic areas of each lesion at 72 h. H&E and GMS staining of adjacent sections are shown below each set of ion maps. Outlines highlight the lesion area on each tissue section. Scale bars, 3 mm.

Given the fact that the standard micafungin regimen in the clinical setting is daily dosing, a multiple-dose experiment was designed to examine drug accumulation of micafungin at steady state. We analyzed drug distribution at 24 h following 2 and 3 therapeutic doses of micafungin. MALDI imaging analysis (Fig. 6a) showed that multidosing had limited impact on partitioning into liver tissue, as drug signals were barely captured in the nonlesion part of tissues even after 3 doses of micafungin. In contrast to marginally detectable drug levels in the surrounding tissues, a noticeably increased drug intensity was observed inside lesions after multiple doses, indicating micafungin was accumulating somewhat within lesion at above-normal tissue drug levels at steady state. Given the extended PK properties of CD101, we were driven to assess whether the quick and favorable lesion penetration after a single dose of CD101 could persist for a long time and/or lead to drug accumulation at least comparable to that of the steady state of micafungin. For this purpose, a single therapeutic dose of CD101 was established in parallel with the multidosing micafungin arm. Tissue samples from the CD101 arm were collected at 48 h and 72 h postdose, equivalent to 24 h after 2 and 3 doses of micafungin, respectively. Consistent with the previous single-dose experiment, at 48 h robust CD101 signal was detected from the entire tissue and drug diffused into lesions more effectively (Fig. 6b). Drug accumulation within the necrotic region of the lesions became more visible at 72 h, when drug intensities in the surrounding tissue were reduced.

### (ii) Quantifying drug levels.

**FIG 7:**
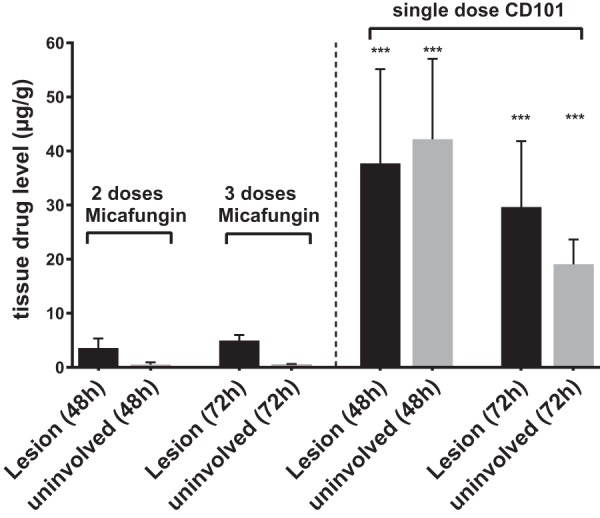
Drug accumulation comparison between multiple doses of micafungin (5 mg/kg) and a single dose of CD101 (20 mg/kg). Absolute drug level was measured for lesions and surrounding uninvolved tissues from liver samples collected at 48 h and 72 h after the first dose of micafungin and those treated with a single dose of CD101 and collected at the matched time points. Error bars indicate means ± SD from 3 to 5 liver pieces or distinct lesions. ***, *P* < 0.001. Drug levels of CD101 were significantly higher than those of micafungin in corresponding sites (lesion or uninvolved tissue) at specified time points.

Compared to a single dose of micafungin, additional daily dosing, which reached steady state after 3 doses, promoted drug retention within lesions even though drug levels in surrounding nonlesion tissues were low, at only ∼0.5 μg/g, and not much different from that at 24 h after single dosing (Fig. 7). Micafungin accumulated in lesions slowly but continuously, retaining 3.5 μg/g and 4.9 μg/g at 24 h after the 2nd and 3rd drug dose, respectively. In comparison, the extensive tissue distribution and lesion penetration after a single dose of CD101 was confirmed once again when samples were assessed at an extended time point to match steady-state micafungin sample collection. Mean CD101 concentrations of 37.7 μg/g and 29.7 μg/g were reported from dissected lesions at 48 h and 72 h, respectively, and corresponding drug levels in surrounding tissues were measured at 42.2 μg/g and 19.1 μg/g (Fig. 7), significantly higher than that of micafungin in corresponding sites at each time point. These results are consistent with the MALDI imaging data.

## DISCUSSION

Despite widespread knowledge that tissue penetration is crucial for the efficacy of antifungal agents, there is no definitive information on the extent to which these drugs penetrate into the site of infection for various invasive fungal infections (28). This important therapeutic issue has been largely ignored experimentally because of long-standing technical obstacles to estimating drug concentration in various tissue subcompartments. The current understanding of tissue distribution of antifungal agents has been based primarily on drug concentration measurement in whole-tissue homogenates (29–32). Drug concentration in tissue homogenates is a useful measure of exposure. However, it can be misleading due to the loss of key information on the spatial distribution of drugs in distinct subcompartments, particularly when abscesses or other forms of lesions are formed. Since infecting organisms often reside within these subcompartments, it would be most valuable if the drug and targeting organism could be colocalized within tissue beds. Here, by employing MALDI imaging technology and LCM-directed drug quantification in a clinically relevant IAC mouse model, we have successfully evaluated for the first time drug exposure within intra-abdominal abscesses.

IAC is difficult to treat, and the outcome is poor even after proper source control and adequate antifungal treatment (2, 4). Moreover, a recent study reported that IAC, particularly cases due to Candida glabrata, was a hidden reservoir for emergence of echinocandin resistance (5). These observations have raised concerns about insufficient drug penetration during therapy for IAC. One of the very few studies examining infection site PK of micafungin reported that there was moderate drug penetration into the peritoneal cavity in IAC patients, with a median area under the concentration-time curve from 0 to 24 h (AUC_0–24_) peritoneal fluid/plasma ratio of 0.3 after the first dose and of 0.3 at steady state (14). Interestingly, our data revealed that micafungin was gradually penetrating into liver and kidney abscesses but only reached detectable levels inside lesions at 6 h after the first dose. The penetration improved upon multiple doses of treatment, and only at steady state were drug signals observed from the necrotic core, where large amounts of fungal cells proliferate. Absolute drug quantification from lesions at different postdose time points further confirmed this drug accumulation pattern.

CD101 is a next-generation echinocandin drug candidate in clinical development. Because of substantially reduced toxicity due to chemical stability and enhanced PK properties, CD101 can be safely dosed at much higher levels than other echinocandin drugs (25, 33, 34). Based on mouse PK and human phase I clinical trial data, 20 mg/kg in mice is considered equivalent to a human therapeutic dose. At this dosing level, CD101 has demonstrated better or at least comparable efficacy *in vivo* against invasive candidiasis relative to micafungin (24). In the present study, CD101 showed extensive tissue distribution with an impressive drug level of 80.1 μg/g in nonlesion parts of liver at 6 h after a single-dose treatment at 20 mg/kg. More notably, the drug was observed to quickly penetrate into abscesses as early as 3 h and rapidly reach the necrotic core, interacting with the main fungal population at 6 h, with an average of 31.6 μg/g drug in lesions. Given the long half-life of CD101 (38.9 h in mice at 20 mg/kg [24]), it is not surprising that sustained drug penetration and accumulation within lesions was continuously observed for all remaining time points included in the study. Even at 72 h following a single dose, drug levels inside lesions were still close to 30 μg/g, about 6-fold higher than that for micafungin at steady state. The outstanding penetration of CD101 at the site of infection is dose dependent. In the low-dose (5 mg/kg) CD101 experiment, we observed the same penetration pattern but proportionally lowered drug levels both in- and outside lesions at selected time points compared to the 20-mg/kg treatment. At 24 h after a single low dose of CD101, the mean drug concentration within lesions was 12.7 μg/g, still about 4-fold higher than what was seen with micafungin (3.4 μg/g) at the same dosage, indicating a true superior lesion penetration feature of CD101.

Drug exposure is one of the most important determinants for efficacy, and we assessed liver burdens in different treatment groups at 6 h and 24 h after a single dose of CD101 or micafungin. Consistent with drug levels, at 6 h postdose, both 20 and 5 mg/kg CD101 had greater burden reduction than 5 mg/kg micafungin (0.7 and 0.9 versus 0.1 log_10_ CFU/g), although the differences were not statistically significant and all treatment groups had two mice with complete resolution of liver infection. Burden reduction efficacy of CD101 was further observed at 24 h with 20-mg/kg treatment, with which 4 of 5 mouse livers were sterilized, which was consistent with accumulating drug in the lesions. In comparison, no liver sterilization was observed for micafungin-treated mice, and resolved liver infection was achieved in one mouse but not others in the 5-mg/kg CD101 group. These data suggest that a prominent drug level (to be defined) at the infection sites is required for resolution of organ invasion in treating IAC with echinocandins. Hence, at the recommended therapeutic dose, currently approved echinocandins may have limited efficacy in IAC treatment.

The ability to quantify kinetically the level of drug at the site of infection has important implications for emergence of drug resistance. Insufficient penetration and/or drug accumulation in lesions may create temporal or spatial windows in specific niches, allowing acquisition of mutations in major drug target genes and eventually facilitating emergence of resistance. The mutant prevention concentration (MPC) derived from the mutant selection window hypothesis, which was raised to address the need for a dosing strategy to restrict emergence of resistance to antibacterial agents (35–37). MPC is the minimal concentration that inhibits drug-susceptible mutant subpopulations. The MPC-based PK/pharmacodynamic measurement, AUC_0–24_/MPC, is suggested to be more accurate than the AUC_0–24_/MIC in predicting resistance occurrence (36, 38, 39), and *in vivo* experiments support its usefulness to control the frequency of resistant mutant development by maintaining drug levels above the MPC for certain periods of time (40, 41). The MPCs for both CD101 and micafungin are reported as 16 μg/ml against wild-type strains of C. albicans and C. glabrata (24). In this study, we found that at steady state micafungin diffused into abscesses at just under 5 μg/g (equal to 5 μg/ml of tissue homogenate), which was above the MIC (MIC of 0.03 μg/ml) but below the reported MPC. This result may help account for echinocandin treatment failures and emergence of resistance observed with some IAC patients, as the level of drug falls below critical levels. In contrast, CD101 penetrated into the lesions as early as 6 h after a single dose at 20 mg/kg, and drug levels were maintained at exceptionally high levels throughout a 72-h endpoint with a mean concentration of 29.7 μg/g (equal to 29.7 μg/ml), which was well above the MPC. A tentative interpretation for this observation is that, under a well-determined and properly designed dosing regimen, CD101 has the potential to overcome or limit resistance development induced by insufficient drug penetration of currently approved echinocandin agents. Nevertheless, studies examining detailed relationships between resistance development and drug penetration and exposure in the context of IAC are warranted.

It should be noted that even with LCM, the usefulness of absolute drug quantification may still be limited when lesions are small (<1 mm^2^). Due to the current limit of detection of LC-MS/MS, we pooled 6 consecutive LCM sections from the same lesion for drug quantification. For small lesions, such a sample pooling method resulted in measuring both inner and outer areas of the lesion rather than just the necrotic core. In this situation, MALDI imaging along with histopathological analyses are the most useful tools to elucidate drug location within lesions. One example is that the average drug concentration of micafungin at steady state within liver lesions was not significantly higher than that at 6 h after single-dose administration. However, MALDI imaging demonstrated remarkably different profiles at these two time points. Drug signals only appeared in the outer layer within the lesion at 6 h after a single dose, whereas fungal hyphae were scattered all over inside the lesion, with the largest amount of cells clumping in the center. In contrast, at steady state, micafungin was mostly seen in the center area of lesions where the majority of the fungal cell population was residing.

In summary, our study represents the first successful application of MALDI imaging in the antifungal field. It opens up a new path to study drug penetration at the site of infection and establishes a cornerstone for advanced antifungal drug research. With this new strategy, we have demonstrated differential spatial distribution of lesion penetration for two echinocandin drugs. The new member of this drug class, CD101, displays extraordinary penetration attributes at the site of infection relative to micafungin. Insights gained in this study have important clinical implications for both treating IAC and preventing emergence of resistance more effectively. Moreover, our findings are potentially relevant to the other predominant type of invasive candidiasis, candidemia, in which seeding of target organs like liver and kidney is also characterized by microabscess formation.

Finally, more broadly, these types of studies are relevant to a wide range of infectious and noninfectious disease pathologies (e.g., tumors) that require effective drug levels for clinical response. It is apparent that some drugs are narrowly dosed to their pharmacodynamic target level, and monitoring serum drug levels may not adequately predict drug exposure at the disease source, especially when necrotic tissue is involved.

## MATERIALS AND METHODS

### Ethics statement.

Mice were housed in the Public Health Research Institute's Animal Biosafety Level-2 Research Animal Facility (ICPH RAF), a center of the New Jersey Medical School, Rutgers University (NJMS-Rutgers). Our animal facility follows the Public Health Service and National Institutes of Health policy of humane care and use of laboratory animals. All experimental protocols were approved by the Rutgers Institutional Animal Care and Use Committee (IACUC).

### Candida albicans strain and antifungal drugs.

C. albicans strain SC4315 was grown in yeast extract-peptone-dextrose broth at 37°C with shaking overnight. Cells were washed, counted, and prepared to 1 × 10^8^ CFU/ml for inoculation as previously described (24). CD101, CD101-D9 (Cidara Therapeutics, Inc., San Diego, CA, USA), and micafungin (Astellas Pharma Inc., Tokyo, Japan) were obtained as standard powders from their manufacturer. [^13^C_6_]micafungin was purchased from Alsachim, France.

### Mouse model of intra-abdominal candidiasis and tissue sample collection.

A mouse model of IAC established by Cheng et al. was used for this study (22). Female 6- to 8-week-old CD1 mice (Charles River Laboratories) weighing 18 to 22 g were infected intraperitoneally (i.p.) with 1 × 10^7^ CFU of C. albicans SC5314 mixed with sterile stool matrix as previously described. Single i.p. doses of CD101 at 20 mg/kg (equivalent to a humanized therapeutic dose) or micafungin at 5 mg/kg (therapeutic dose) were administered to groups of 15 mice at day 3 postinoculation. Mice were sacrificed just before antifungal treatment (*n* = 1) and at 1, 3, 6, 24, and 48 h postdose (3 mice per group per time point). Livers and kidneys were explored for abscesses of >1 mm in diameter (Fig. 1), dissected, placed on a cryohistology tray, snap-frozen in liquid nitrogen, and stored at −80°C for tissue sectioning for MALDI imaging. We also performed an experiment where a low dose of CD101 at 5 mg/kg was administered and micafungin was given at the same dose. Liver and kidney samples were collected at 6 h and 24 h postdose for both MALDI imaging and absolute drug quantification. Moreover, infected livers were collected from 45 mice (5 mice per time point per group) and measured for burden counts at 0, 6, and 24 h after single-dose treatment of 20 or 5 mg/kg CD101, 5 mg/kg micafungin, or vehicle control. Treatment was started at day 3 postinfection for all groups. In another separate experiment that aimed at comparing therapeutic levels of a single dose of CD101 and multiple doses of micafungin, a single dose of CD101 at 20 mg/kg was given at day 3 postinoculation, and once-daily treatment of micafungin at 5 mg/kg started from day 3 postinoculation. A total of 3 doses of micafungin was administered. Livers and kidneys were collected at 48 and 72 h after the first dose of each drug.

### Tissue sectioning and matrix application.

Tissues were sectioned at 12-μm thickness using a Leica CM1850 cryostat (Buffalo Grove, IL) and mounted onto stainless steel slides (for MALDI-MSI analysis) or frosted glass microscope slides (for H&E staining) as previously described (42). Tissue sections were stored at −80°C until analysis. Prior to MALDI-MSI analysis, tissue sections were thawed and exposed to ionization matrix. For CD101, 2,5-dihydroxybenzoic acid (DHB) (20 mg/ml in 50% methanol) containing 267 fmol/μl CD101-D9 (Cidara Therapeutics, Inc.) was applied to the surface using an HTX TM-sprayer (Chapel Hill, NC) operating with a 50-μl/min flow rate, 60°C nozzle temperature, and 5 lb/in^2^ of pressure. Twenty-five passes over the tissue were performed. For micafungin, 1,5-diaminonaphthalene (1,5-DAN) matrix (5 mg/ml in 50% acetone) containing 5 nmol/μl [^13^C_6_]micafungin (Alsachim, France) was coated onto tissue surfaces by the TM-sprayer at a flow rate of 60 μl/min, 50°C nozzle temperature, and 5 lb/in^2^ of pressure. Twenty-five passes over the tissue were performed.

### MALDI-MSI analysis.

MALDI-MSI analysis was performed using a MALDI LTQ Orbitrap XL mass spectrometer (Thermo Fisher Scientific, Bremen, Germany) with a resolution of 60,000 at *m/z* 400, with full width at half maximum. The resolution was sufficient to resolve CD101, CD101-D9, micafungin, and [^13^C_6_]micafungin peaks from the background without the requirement of MS/MS and subsequent loss of signal. However, drug peak identities were confirmed by acquiring several MS/MS spectra directly from the dosed tissues. Standards of CD101 and micafungin were analyzed directly from the stainless steel target plate and spiked into drug-naive liver tissue to optimize instrument parameters. The limit of detection (LOD) for MALDI-MSI analysis of micafungin and CD101 was 500 ng/g and 1 μg/g of liver or kidney tissue, respectively, calculated as described previously (19).

For CD101, spectra were acquired in the *m/z* 1,000 to 1,500 range, using positive ionization with a laser energy of 25 μJ, and 15 laser shots were fired at each position. Spectra for micafungin were acquired in the same *m/z* range under a negative ionization mode with a laser energy of 10 μJ and 5 laser shots at each position. The laser step size was set at between 50 and 75 μm, a size at which small necrotic areas within lesions could easily be resolved, and no overlapping of the laser spot on adjacent acquisitions was observed.

Data visualization was performed using Thermo ImageQuest software. Normalized ion images of CD101 were generated by dividing CD101 [M+H]^+^ signal (*m/z* 1,225.603 ± 0.005) by CD101-D9 [M+H]^+^ signal (*m/z* 1,234.651 ± 0.005). Normalized ion images of micafungin were generated by dividing micafungin [M+H]^−^ signal (*m/z* 1,268.444 ± 0.005) by [^13^C_6_]micafungin [M+H]^−^ signal (*m/z* 1,274.455 ± 0.005).

### Laser-capture microdissection.

Necrotic lesion and surrounding tissue areas totaling 2 × 10^6^ to 6 × 10^6^ μm^2^ were dissected from between 3 and 6 serial liver or kidney biopsy tissue sections using a Leica LMD6500 system (Buffalo Grove, IL). Lesion areas were identified optically from the brightfield image scan and by comparison to the adjacent sectioned H&E reference tissue. Pooled dissected lesion tissues were collected into 0.25-ml standard PCR tubes and immediately transferred to the −80°C freezer for storage.

Prior to analysis, the tubes were thawed at room temperature for 30 min. Fifty microliters of extraction solution (acetonitrile-methanol [ACN-MeOH; 1/1] with 100 ng/ml CD101-D9 and 100 ng/ml [^13^C_6_]micafungin) was added to each tube, which were then sonicated for 5 min and centrifuged at 10,000 rpm for 5 min at room temperature. Forty microliters of supernatant was transferred for LC-MS/MS analysis and diluted with an additional 40 μl of MilliQ water.

Neat 1-mg/ml dimethyl sulfoxide (DMSO) stocks for all compounds were serially diluted in 50:50 acetonitrile-water to create standard curves and quality control spiking solutions. Three microliters of neat spiking solutions was added to 2 μl of lesion homogenate, and extraction was performed by adding 50 μl of extraction solution (ACN-MeOH [1:1] with 100 ng/ml CD101-D9 and 100 ng/ml [^13^C_6_]micafungin). Extracts were vortexed for 5 min and centrifuged at 10,000 rpm for 5 min. A 40-μl aliquot of supernatant was transferred for LC-MS/MS analysis and diluted with an additional 40 μl of MilliQ water. Previously optimized LC-MS/MS parameters were used for analysis (see the next section).

### Drug quantitation by LC-MS/MS.

LC-MS analysis was performed on a Q Exactive high-resolution mass spectrometer (Thermo Fisher Scientific, Waltham, MA) coupled to a Thermo Scientific Dionex UltiMate 3000 binary system. Chromatography was performed with a Kinetex C_18_ column (2.1 by 50 mm; particle size, 1.7 μm; Phenomenex, Torrance, CA) using a reverse-phase gradient elution, ACN-H_2_O (60:40) and 10 mM ammonium acetate for mobile phase A, and 2-propanol (IPA)-ACN-MeOH (80:10:10) and 10 mM ammonium acetate for mobile phase B. A flow rate of 300 μl/min was used, with a gradient consisting of 20% B held for 0.5 min, followed by linear increase to 95% in 3.5 min, held for 2.2 min, and a return to the initial 20% B in 0.3 min. The column was equilibrated for 1.5 min before the next injection, and the temperatures of the column and sample tray were held at 50 and 4°C, respectively. The column retention times for CD101 and micafungin were 3.32 min and 3.2 min, respectively.

Key MS parameters were the following: spray voltage, 3.5 kV; capillary temperature, 320°C; heated electrospray ionization (HESI) probe temperature, 400°C; S-lens radio frequency level, 50. The sheath gas and auxiliary gas were set to 45 and 10 U, respectively. External mass calibration was performed before each sequence. For CD101, a full scan was applied in positive ionization mode with a mass range of *m/z* 250 to 1,500 at a resolution power of 70,000, with an automatic gain control target of 3e6 for a maximum injection time (IT) of 100 ms. For micafungin, a full scan was applied in negative ionization mode with a mass range of *m/z* 250 to 1,500 at a resolution power of 70,000 and AGC target of 3e6 for a maximum IT of 100 ms. CD101 [M+H]^+^ signal was normalized to CD101-D9 [M+H]^+^ signal, and micafungin [M+H]^−^ signal was normalized to [^13^C_6_]micafungin [M+H]^−^ signal.

### Statistical analysis.

Absolute drug concentrations were graphed and statistically analyzed in GraphPad software (Prism 7; GraphPad Software, Inc., San Diego, CA). Drug levels in different tissue compartments at different time points were compared by one-way analysis of variance (ANOVA), and Dunn's multiple comparison was used for the *post hoc* analyses. Statistical significance was defined as a *P* value of <0.05. Prompt antifungal therapy and source controls are crucial for successful treatment.

## Supplementary Material

Supplemental material
