# Supplementary material for: Unraveling Drug Penetration of Echinocandin Antifungals at the Site of Infection in an Intra-abdominal Abscess Model
Source: Antimicrob Agents Chemother. 2017 Sep 22;61(10):e01009-17. doi: 10.1128/AAC.01009-17 (PMC5610477; doi:10.1128/AAC.01009-17)
Supplement: Supplemental material [file supp_61_10_e01009-17__index.html]

Supplemental material 

# Unraveling Drug Penetration of Echinocandin Antifungals at the Site of Infection in an Intra-abdominal Abscess Model

## Supplemental material

- Supplemental file 1 -

  Supplemental Figures S1 and S2

  PDF, 1.1M
