# Supplementary material for: Unraveling Drug Penetration of Echinocandin Antifungals at the Site of Infection in an Intra-abdominal Abscess Model
Source: Antimicrob Agents Chemother. 2017 Sep 22;61(10):e01009-17. doi: 10.1128/AAC.01009-17 (PMC5610477; doi:10.1128/AAC.01009-17)
Supplement: Supplemental material [file AAC.01009-17_zac010176564s1.pdf]

Scalebar = 0.5mm

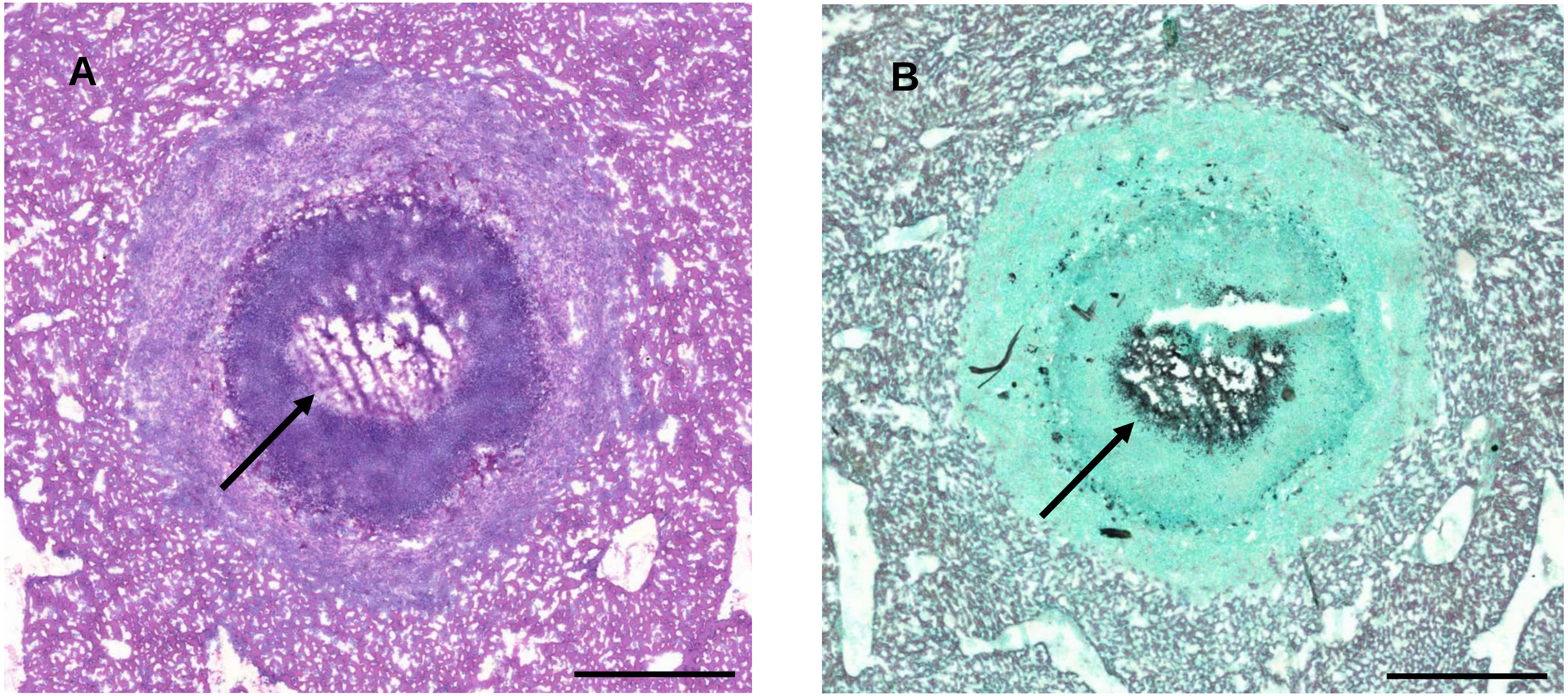

Supplementary Figure 1. Enlarged H&E and GMS staining of one liver lesion. Liver lesions are characterized by densely-packed neutrophils surrounding a highly necrotic acellular core (black arrow shown in panel A). GMS stain of fungi distribution (black arrow shown in Panel B) correlates with highly necrotic center of lesion.

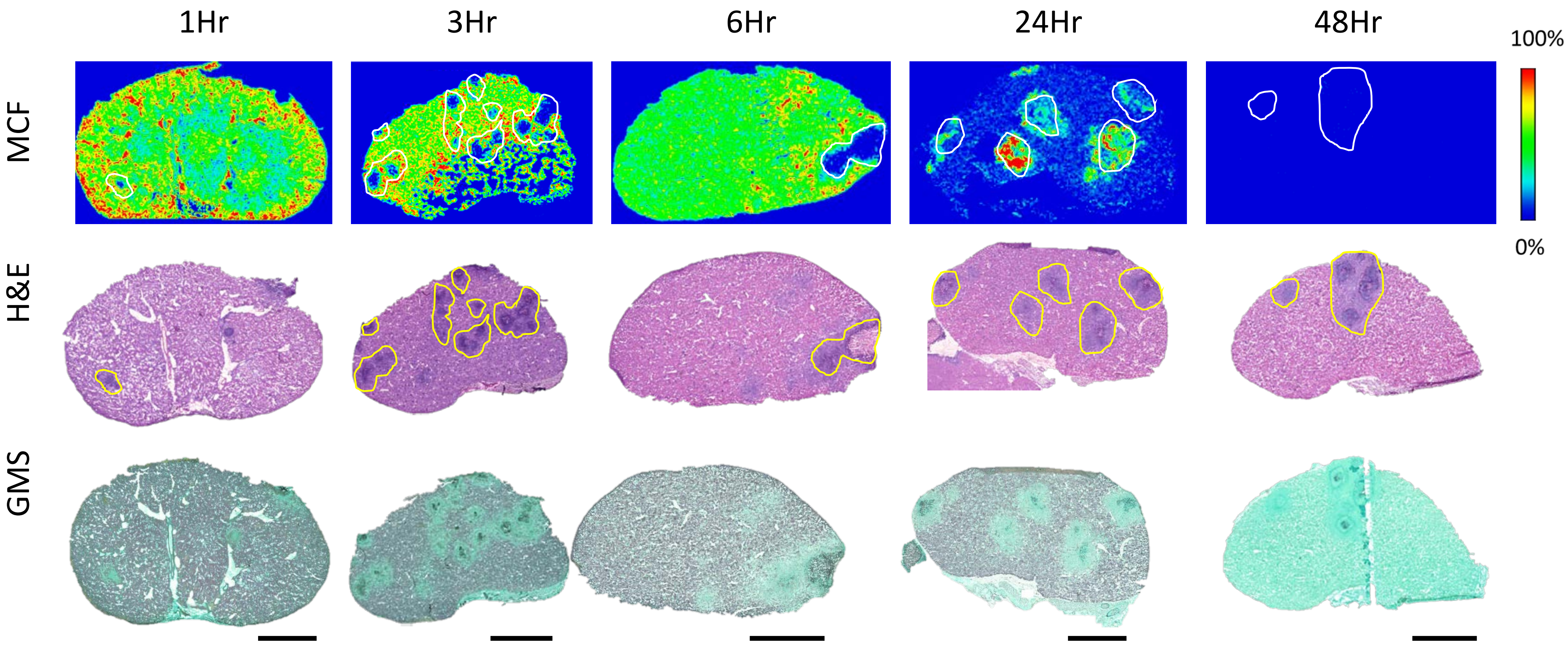

Supplementary Figure 2. Drug distribution in infected kidneys post single dose micafungin and CD101. **(a)** Upper row: Ion maps of micafungin in representative kidney tissues collected at 1, 3, 6, 24, and 48h post a single dose of micafungin at 5 mg/kg; signal intensity color bar is fixed for micafungin, with gradually increased intensity from blue (no signal) to red (max signal); H&E and GMS staining of adjacent sections are shown below each set of ion maps. Outlines highlight the lesion area on each tissue section. Scale bars, 3 mm.

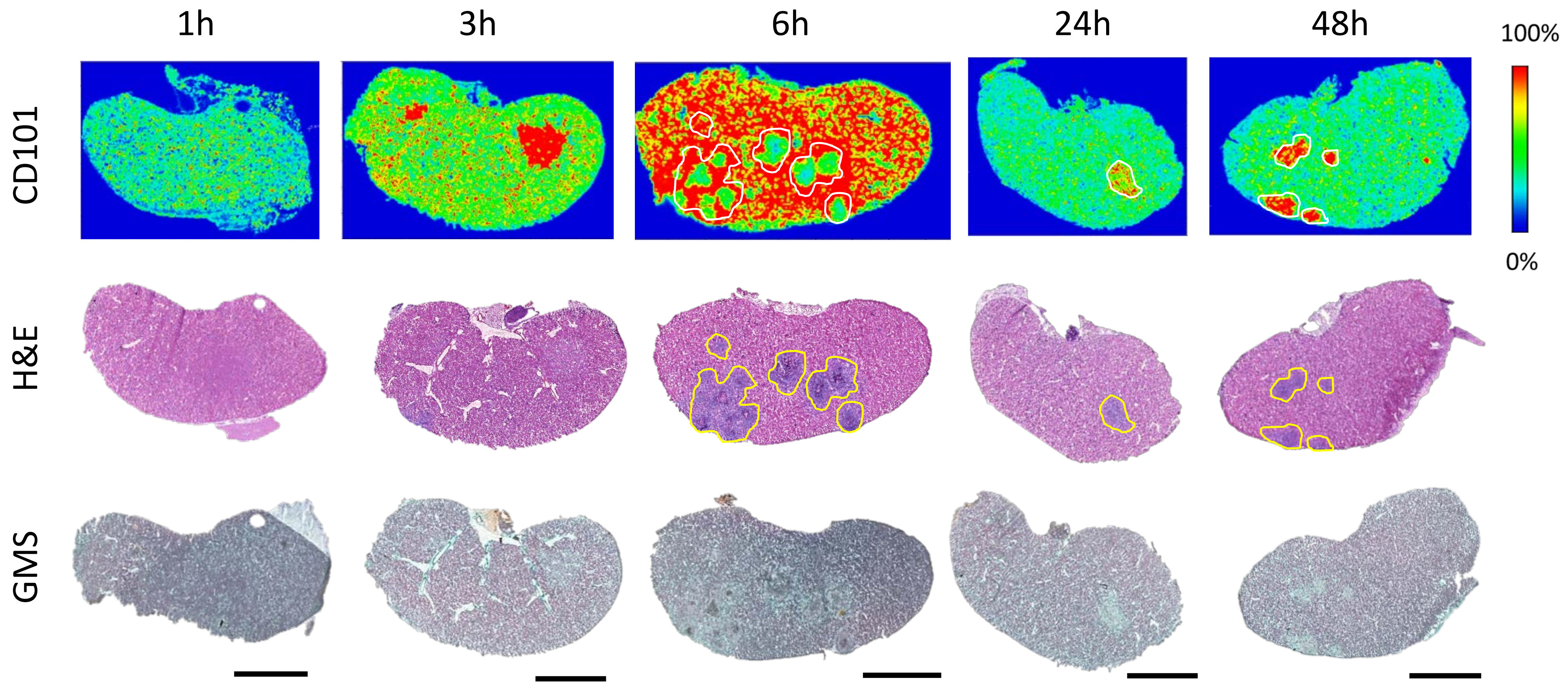

Supplementary Figure 2b. Upper row: Ion maps of CD101 in representative kidney tissues collected at 1, 3, 6, 24, and 48h post a single dose of CD101 at 20 mg/kg; signal intensity color bar is fixed for CD101, with gradually increased intensity from blue (no signal) to red (max signal). Matched H&E and GMS staining results are shown in the middle and bottom rows, respectively. Scale bars, 3 mm.
